# Effect of a single exercise bout on fasting cerebral blood flow and brain insulin sensitivity in middle‐aged to older adults

**DOI:** 10.1113/EP093333

**Published:** 2025-12-24

**Authors:** Steven K. Malin, Ankit M. Shah, Michal S. Berri, David H. Zald, Daniel J. Battillo

**Affiliations:** ^1^ Department of Kinesiology and Health Rutgers University New Brunswick New Jersey USA; ^2^ Department of Medicine, Division of Endocrinology, Metabolism and Nutrition Rutgers University New Brunswick New Jersey USA; ^3^ New Jersey Institute for Food, Nutrition and Health Rutgers University New Brunswick New Jersey USA; ^4^ Institute of Translational Medicine and Science Rutgers University New Brunswick New Jersey USA; ^5^ Institute for Health, Health Care Policy and Aging Research Rutgers University New Brunswick New Jersey USA; ^6^ Center for Advanced Human Brain Imaging Research Rutgers University Piscataway New Jersey USA

**Keywords:** cognition, dementia, insulin resistance, obesity, type 2 diabetes

## Abstract

Reductions in brain insulin sensitivity and cerebral blood flow (CBF) have emerged as potential factors contributing to Alzheimer's disease and related dementia. However, no work has tested whether a single bout of exercise can raise brain insulin sensitivity in at‐risk adults. The aim of the study was to test whether a single bout of exercise raises brain insulin sensitivity in middle‐aged to older adults with cardiometabolic risk. In a counterbalanced pilot study design, 15 cognitively unimpaired (Montreal Cognitive Assessment, 28.2 ± 1.3 a.u.) adults [56.7 ± 2.1 years old; maximal oxygen consumption (${\dot{V}}_{O_{2}\max}$), 23.9 ± 0.9 mL/kg/min] with excess body weight (body mass index, 31.8 ± 1.3 kg/m^2^) and impaired glucose tolerance (haemoglobin A1c, 5.8% ± 0.30%) were randomized to a rest (time‐matched control) or acute treadmill exercise bout (70% ${\dot{V}}_{O_{2}\max}$ for 60 min) in the evening. The next morning, participants arrived fasted to determine brain insulin sensitivity. Plasma glucose and insulin, in addition to CBF, were assessed by pseudo‐continuous arterial spin labelling before and after intranasal insulin (40 IU). Cognition (NIH toolbox), aerobic fitness (${\dot{V}}_{O_{2}\max}$) and body composition (dual‐energy X‐ray absorptiometry) were also analysed. There was no difference in plasma glucose or insulin following intranasal with or without exercise. Although cognition was also not improved, exercise increased CBF in the hippocampus, putamen and pallidum (condition effect, *P* < 0.05). Exercise‐induced increases in fasting hippocampus, caudate, pallidum and putamen CBF were correlated with a lowering of CBF responses to insulin. In middle‐aged to older adults with cardiometabolic risk, a single bout of exercise increased fasting CBF, and this related to decreased CBF insulin responses in regions related to memory and motor control.

## Abstract (highlights)

- **What is the central question of this study?**
  Does a single bout of exercise the night before impact cerebral blood flow (CBF) and brain insulin sensitivity the next morning?
- **What is the main finding and its importance?**
  Our results show that a single bout of exercise increases fasting putamen, pallidum and hippocampus CBF in middle‐aged to older adults with cardiometabolic risk. Although there was no statistical effect of intranasal insulin on CBF, exercise‐induced increases in fasting caudate, pallidum, putamen and hippocampus CBF were related to lower CBF responses to insulin. These preliminary findings suggest that acute exercise might enhance CBF.

## 1 INTRODUCTION

Cerebrovascular dysfunction and insulin signalling deficiency are two key contributors to cognitive impairment in middle‐aged to older adults (Arnold et al., 2018; Eisenmenger et al., 2023; Leclerc et al., 2022). Brain insulin action is a logical target for intervention, given that insulin receptors are densely localized in the hippocampus and cortical areas (primarily in synapses; Leclerc et al., 2022) of the brain (Katsel et al., 2018). Higher concentrations of vascular insulin receptors are correlated with better cognitive function, whereas cerebrovascular insulin resistance is seen in the Alzheimer's disease brain (Arnold et al., 2018).

Currently, only one study has compared the effects of insulin on cerebral blood flow (CBF) in older and younger adults, reporting elevated intranasal insulin (INI)‐stimulated thalamic CBF in older adults versus no change in young adults (Akintola et al., 2017). These findings are consistent with work showing that ageing adults, with or without hyperglycaemia, often have decreased fasting brain glucose metabolism and CBF, suggesting that the increase in CBF/glucose metabolism seen with INI might reflect a compensatory mechanism (Arnold et al., 2018).

Further complicating this matter is that the progression from middle age to late adulthood is also associated with cardiometabolic and cognitive decline risk (Bendlin et al., 2010; Gottesman et al., 2017). Patients with normal weight, for instance, show decreased CBF in response to INI in the frontal gyrus, prefrontal cortex and hypothalamic regions compared with no response or increased CBF responses in those with obesity (Kullmann et al., 2018; Wingrove et al., 2019, 2021). Moreover, not only does having type 2 diabetes nearly double the risk for progression from mild cognitive impairment to Alzheimer's disease and related dementia (Cooper et al., 2015; Pal et al., 2018), but others report that even prediabetes can inhibit the reversal from mild cognitive impairment to normal cognition (Makino et al., 2021). Thus, work is needed to understand how treatments might impact fasted and insulin‐stimulated CBF among ageing adults with cardiometabolic risk (e.g., obesity/hyperglycaemia).

Aerobic exercise is associated with reduced risk of dementia (Malin et al., 2022; Ogino et al., 2019). The recent EXERT trial highlights cognitive preservation in individuals with mild cognitive impairment undergoing either exercise or stretching interventions, although contrasting results in the LIFE trial exist (Sink et al., 2015). Both single bouts and chronic exercise improve cerebrovascular function (Lake et al., 2022; Ogoh et al., 2005; Palmer et al., 2023; Steventon et al., 2020), including resting CBF, in cognitively normal older adults (Palmer et al., 2023; Tomoto et al., 2023) and those with mild cognitive impairment (Tomoto et al., 2021). Exercise also improves blood–brain barrier (BBB) integrity for insulin delivery and neuronal insulin signalling in rodents that collectively favour cognitive functions (Brown et al., 2022; Marosi et al., 2012; Park et al., 2019; Ruegsegger et al., 2019). Although exercise in humans increases insulin sensitivity in skeletal muscle, vascular and fat tissues (Heiston & Malin, 2019; Heiston, Gilbertson et al., 2021; Heiston, Liu et al., 2021; Heiston et al., 2022; Malin et al., 2016), it is unclear whether exercise improves brain insulin sensitivity. In the only study to date, 8 weeks of exercise increased fasting cerebellum CBF and INI‐induced CBF in the striatum (i.e., putamen) and INI‐stimulated hippocampal functional connectivity (Kullmann et al., 2022) among young, overweight adults with normoglycaemia. Although these findings together suggest that exercise might contribute to regions influencing memory, learning and motor control, this prior work used an insulin dose of 160 IU, which is commonly used for mechanistic investigation but is not used clinically owing to concern of potential insulin spillover into the periphery and hypoglycaemia risk, particularly if used before exercise (Gwizdala et al., 2021). Whether exercise influences INI‐stimulated CBF in hippocampus and motor control regions using a clinical INI dose prior to weight loss or increased fitness is unknown.

Previously, we reported that a single bout of exercise performed the night before increased peripheral vascular insulin sensitivity in middle‐aged adults with obesity (Heiston, Liu et al., 2021). Given the effect of this exercise on peripheral vascular insulin sensitivity, we hypothesized that these peripheral gains in insulin sensitivity extend to the brain. Thus, we tested the hypothesis that a single bout of exercise performed the night before would increase fasting CBF and increase brain insulin responses to a clinical INI dose of 40 IU in ageing adults.

## 2 MATERIALS AND METHODS

### 2.1 Ethics approval

Study protocols conform to the *Declaration of Helsinki* and were approved by our Institutional Review Board (IRB no. 2022001842). All participants provided written and verbal consent for this pilot study. The overall study was registered on Clinicaltrials.gov (NCT no. 05853913).

### 2.2 Study design and participants

Adults aged 40–80 years with overweight or obesity (body mass index, 25–45 kg/m^2^) were recruited for this randomized, counterbalanced study via paper advertisements, social media and electronic medical records from the New Brunswick, NJ, USA area. Individuals were included in the study if non‐smoking, physically inactive (<150 min/week of moderate‐intensity exercise), weight stable within 2 kg over the last 3 months, not taking insulin for glycaemic control, and free of chronic disease (e.g., renal, hepatic, cardiovascular). Fasting blood chemistries (e.g., complete blood count, haemoglobin A1c, lipid panel, comprehensive metabolic panel) were assessed for normality during the screening to ensure study eligibility. A 12‐lead ECG was completed during rest and the maximal aerobic exercise test to monitor heart rhythms. Blood chemistries and ECGs were assessed and cleared by the study medical doctor in conjunction with a physical examination. Individuals were also screened for mild cognitive impairment using the Montreal Cognitive Assessment, and those scoring >25 (i.e., free of cognitive impairment) were included in the study. One female with a self‐reported regular menstrual cycle completed the study clinical visits ∼1 month apart in the follicular phase. No women took oral contraceptives or hormone replacement therapy.

### 2.3 Body composition and fitness

Total body weight was recorded using a digital scale, with participants wearing minimal clothing and shoes removed. Waist circumference was measured in duplicate using a tape measure ∼2 cm above the umbilicus and averaged for recording. Height was measured to the nearest 0.1 cm using a stadiometer. Total body fat and lean body mass were assessed via dual‐energy X‐ray absorptiometry (GE Healthcare Lunar, Madison, WI, USA). Maximal oxygen consumption (${\dot{V}}_{O_{2}\max}$) was assessed via a continuous, incremental treadmill test using indirect calorimetry prior to any test visit using standard criteria (Heiston, Liu et al., 2021).

### 2.4 Condition visits

Participants completed resting (control) and exercise conditions in a randomized, counterbalanced order on the night prior to each clinical testing visit. The visits were completed between 17:00 and 20:00 h, and there was a washout period of ≥1 week (≤1 month for the premenopausal woman) between conditions. The exercise condition consisted of 5 min of resting indirect calorimetry to establish baseline measures, followed by a 5 min warm‐up (45% ${\dot{V}}_{O_{2}\max}$) and 60 min of treadmill walking at 70% ${\dot{V}}_{O_{2}\max}$. Heart rate, oxygen consumption, respiratory exchange ratio and rating of perceived exertion were recorded every 5 min. The resting conditions consisted of 60 min of supervised seated rest to mimic exercise conditions. Participants were provided with standardized (∼55% carbohydrate, 30% fat and 15% protein) breakfast and lunch meals during the day of each visit and instructed to eat their standardized dinner meal at home after completion of the visit. The timing of dinner consumption was matched between conditions.

### 2.5 Metabolic controls

Total daily energy expenditure was estimated from fasting indirect calorimetry by assessing resting metabolic rate and multiplying it by an activity factor of 1.2. Food logs assessing the timing and extent of meal consumption were administered to the participant and replicated between both conditions. Participants were then instructed to refrain from caffeine, alcohol and medication for 24 h prior to testing. Sleep was documented via a diary, and no differences were noted between conditions (rest, 7.14 ± 1.09 h vs. exercise, 7.11 ± 1.52 h, *P* = 0.93). Participant testing was done at the same approximate time of day between conditions to minimize diurnal variation influences.

### 2.6 Brain imaging and intranasal insulin administration

Participants arrived at the Center for Advanced Human Brain Imaging Research (CAHBIR) after a 10–12 h fast following the experimental conditions, in alignment with prior work from our laboratory (Heiston, Liu et al., 2021). Upon arrival, the participant was placed in an upright chair in a quiet room for 5 min, and an intravenous line was placed for obtainment of fasting glucose and insulin. Cognition was then assessed using the NIH toolbox on an iPAD. The cognitive battery included: (1) episodic memory (Picture Sequence Memory Test; ∼10 min); (2) working memory (List Sorting; ∼7 min); (3) executive function (Flanker Inhibition Test; ∼4 min); and (4) processing speed (Pattern Comparison; ∼3 min). Learning effects were minimized for memory tasks through use of different sequences offered according to NIH manual recommendations.

Thereafter, participants were moved to the MRI room 14.67 ± 0.69 h after exercise, and using a 3 T Siemens Prisma, they received an MPRAGE T1 structural image (1 mm isotropic resolution) for spatial normalization and definition of the regions of interest (Trotier et al., 2022). To measure CBF, participants underwent a pseudo‐continuous arterial spin labelling sequence with a single post‐labelling delay (Time to Echo = 31.8 ms, Time of Repetition = 4060 ms, labelling duration = 1509 ms, with 40 3‐mm‐thick slices and 3.75 mm^2^ in‐plane resolution for whole brain coverage).

Following these fasting images, participants were moved outside the MRI room and administered INI at a dose of 40 IU of human insulin (0.4 mL; Humulin, Eli Lilly & Co., Indianapolis, IN, USA). Single puffs of 0.1 mL occurred four times (twice in each nostril) over 2 min via the Vianase electronic atomisers (Kurve Technology Inc., Lynnwood, WA, USA). This insulin dose was selected for two main reasons. First, 40 IU is used commonly for clinical purposes, as demonstrated recently in the MemAID trial, reporting that this INI therapy raised fasting CBF, cognition and physical function (i.e., 6 min walk test) and decreased peripheral insulin resistance (i.e., HOMA‐IR) and weight (Novak et al., 2022). Second, higher doses of INI used prior to exercise relate to hypoglycaemia risk (Gwizdala et al., 2021).

After 30 min, a blood sample was obtained again, and the scanning was repeated 15.75 ± 0.70 h after exercise to determine post‐INI CBF to determine brain insulin sensitivity. Brain insulin sensitivity was defined as the change from fasting to INI (e.g., CBF_INI_ − CBF_fast_), as done by others (Kullmann et al., 2022). Final blood measures were obtained after imaging (i.e., 90 min), and the total area under the curve using the trapezoidal model was calculated to identify potential spillover effects.

CBF was calculated with ASL‐prep, which performs susceptibility distortion correction, a motion correction, slice‐timing correction and computes CBF using a one‐compartment model and extracts CBF based on regions of interest from co‐registered atlas templates following spatial regularization partial volume correction (BASIL) (Adebimpe et al., 2022). Analysis of CBF focused on the hippocampus as the primary outcome defined by regions of interest from Human Connectome Project (HCP) subcortical atlas (Huang et al., 2022). Based on the finding that a sustained exercise programme increased fasting cerebellar CBF and INI‐induced CBF in the striatum (right putamen) (Kullmann et al., 2022), we also assessed CBF in the cerebellum, putamen and caudate nucleus, in addition to the neighbouring pallidum.

### 2.7 Statistical analysis

Given that in our prior work on vascular insulin sensitivity we enrolled *n* = 12 people (Heiston et al., 2021), we enrolled 15 here for pilot testing on INI‐stimulated CBF. With *n* = 15, the Clopper Pearson exact 95% confidence intervals (CIs) for a targeted enrolment rate, intervention acceptability rate and power of 0.80 range from (0.444, 0.975) and (0.563, 0.943), respectively. These CIs were narrow enough to ensure medium estimated effect rates (Teresi et al., 2022). Data were analysed using the software R (The R Foundation, Vienna, Austria, 2013). Fasting outcomes were compared between conditions using Student's two‐tailed paired *t‐*test. A repeated‐measures ANOVA was used to assess differences between the resting and exercise conditions over time. Condition and time were considered fixed variables, while the participants were the random variable. Insulin‐stimulated CBF data were compared by ANOVA and co‐varied for fasting CBF. Pearson correlations were used to assess ageing between outcomes, in addition to the change in both fasting and INI‐stimulated CBF (i.e., exercise minus rest conditions). Significance was accepted as *P* ≤ 0.05, and trends were accepted as *P* > 0.05 to *P* ≤ 0.10 given the pilot nature of the study. Effect sizes were calculated to assess the physiological relevance among condition differences, with Cohen's *d* for Student's paired *t*‐tests interpreted as small *d* = 0.2, medium *d* = 0.5 and large *d* = 0.8, and partial eta squared for repeated‐measures ANOVAs interpreted as small η^2^ = 0.01, medium η^2^ = 0.06 and large η^2^ = 0.14. Data are the means ± SD.

## 3 RESULTS

### 3.1 Participant and acute exercise characteristics

Participants were middle‐aged, with obesity, mild hyperglycaemia and low aerobic fitness (Table 1). All participants performed a single bout of exercise at moderate to high intensity (69.4% ± 1.0% ${\dot{V}}_{O_{2}\max}$) and expended an average of 412.3 ± 24.4 kcal over the duration of the exercise session (Table 1).

**TABLE 1:** Participant characteristics.

Demographics	Value	Range	95% Confidence interval
*n*	15 (12 female)	–	–
Non‐Hispanic White	12	–	–
Non‐Hispanic Black	1	–	–
Hispanic	2	–	–
Asian Pacific Islander	0	–	–
Age, years	56.7 ± 8.2	41–71	52.46–60.86
Education (degree)			
College/bachelors	8	–	–
Masters	6	–	–
PhD	1	–	–
Handedness (right)	14	–	–
Family history of dementia, %	8 (53)	–	–
MoCA, a.u.	28.2 ± 1.3	26.0–30.0	27.53–28.8
Body composition			
Weight, kg	86.8 ± 3.9	67.5–116.0	78.73–94.3
BMI, kg/m^2^	31.8 ± 5.0	25.0–40.0	29.15–34.2
Body fat, %	44.3 ± 7.2	33.4–56.6	40.66–48.0
Visceral fat, kg	1.5 ± 0.6	0.3–2.5	1.16–18.8
LBM, kg	44.5 ± 7.4	35.3–63.5	42.1–49.6
Fitness and submaximal exercise			
${\dot{V}}_{O_{2}\max}$, mL/kg/min	23.9 ± 3.5	19.1–32.1	22.1–25.6
% ${\dot{V}}_{O_{2}\max}$	69.4 ± 1.0	63.0–79.4	67.4–71.3
% HR_max_	81.0 ± 1.5	70.2–90.3	77.8–84.1
EE, kcal	412.3 ± 24.4	265.7–641.2	364.3–460.2
Cardiometabolic risk			
Fasting blood glucose, mg/dL	103.6 ± 10.6	84–116	95.75–106.51
Haemoglobin A1c, %	5.8 ± 0.3	5.4–6.4	5.65–5.96
Triglycerides mg/dL	123.53 ± 52.69	41–225	96.86–150.19

*Note*: Data are the mean ± SD. Data reflect *n* = 15.

Abbreviations: BMI, body mass index; EE, energy expenditure; LBM, lean body mass; MoCA, Montreal cognitive assessment; ${\dot{V}}_{O_{2}\max}$, maximal oxygen consumption.

### 3.2 Glucose and insulin

There were no differences in fasting plasma glucose (*P* = 0.36) or insulin (*P* = 0.93) between rest and exercise conditions (Table 2). Furthermore, plasma glucose and insulin did not change throughout the protocol, as reflected by the total area under the curve (Table 2). Importantly, we experienced no hypoglycaemic effects (<70 mg/dL) in any participant after INI in either of the conditions.

**TABLE 2:** Fasting and post‐intranasal insulin biochemistry and cognition.

Parameter	Rest	Exercise	*P*‐value	Cohen's *d*
Glucose, mg/dL				
Fasting, mg/dL	98.9 ± 15.7	100.7 ± 17.4	0.36	0.24
tAUC_0–INI90min_	8718 ± 1373.5	8672.4 ± 1443.5	0.27	0.30
Insulin, uU/mL				
Fasting	11.0 ± 6.7	11.1 ± 7.3	0.93	0.02
tAUC_0–INI90min_	833.6 ± 429.2	865.6 ± 475.3	0.71	0.10
Cognition, T‐scores				
PSMT	64.0 ± 14.5	59.9 ± 11.8	0.86	0.12
Flanker	47.7 ± 9.9	49.8 ± 10.7	0.51	0.23
Pattern comparison	64.8 ± 13.9	62.1 ± 18.8	0.49	0.13
List sorting	62.0 ± 9.9	61.4 ± 10.4	0.71	0.04

*Note*: Data are the mean ± SD. Glucose and insulin data reflect *n* = 15. Cognition data are fully corrected T‐scores and reflect *n* = 14.

Abbreviations: Flanker, Flanker Inhibitory Control Attention Test; INI, intranasal insulin; List Sorting, List Sorting Working Memory Test; Pattern Comparison, Pattern Comparison Processing Speed Test; PSMT, Picture Sequence Memory Test; tAUC, total area under the curve.

### 3.3 Cognition and CBF

Although there were no effects of exercise on cognition in these participants (Table 2), exercise increased CBF in the putamen, pallidum and hippocampus, with medium to large effects (condition effect, *P* < 0.05; η^2^ = 0.10–0.15; Table 3). The caudate nucleus trended to increase and had medium effect sizes (η^2^ = 0.07–0.08), but it was not statistically significant (Table 3). These observations were probably driven by changes in fasting CBF, because only INI‐stimulated pallidum CBF tended to increase with medium effect sizes (left, *P* = 0.057 and right, *P* = 0.054, both *d* = 0.59; Table 4).

**TABLE 3:** Cerebral blood flow before and after intranasal insulin with and without exercise.

	Rest		Exercise					
Brain region	0 min	INI 30 min	0 min	INI 30 min	Condition	Time	*C* × *T*	η^2^
Left region, mL/min								
Hippocampus	81.66 ± 38.85	76.13 ± 33.59	90.11 ± 34.92^†^	93.71 ± 35.87^†^	<0.01	0.84	0.34	0.15
Caudate	40.84 ± 19.91	44.62 ± 20.74	47.96 ± 18.97	50.66 ± 19.12	0.055	0.94	0.87	0.08
Pallidum	77.27 ± 37.99	65.52 ± 36.32	79.48 ± 36.20^†^	80.92 ± 31.85^†^	0.03	0.21	0.11	0.10
Putamen	78.50 ± 37.03	71.94 ± 31.74	85.91 ± 31.32^†^	88.44 ± 28.96^†^	0.01	0.66	0.33	0.14
Cerebellum	76.56 ± 35.90	65.17 ± 34.19	75.52 ± 32.88	73.67 ± 38.39	0.45	0.18	0.34	0.01
Right region, mL/min								
Hippocampus	81.99 ± 40.55	80.24 ± 37.85	93.90 ± 40.59*^†^	92.99 ± 41.87^†^	0.01	0.77	0.92	0.14
Caudate	42.30 ± 19.94	46.75 ± 23.43	51.11 ± 22.24	49.83 ± 22.43	0.08	0.63	0.39	0.07
Pallidum	74.73 ± 33.89	74.00 ± 33.95	74.87 ± 39.86	79.05 ± 32.77	0.58	0.71	0.60	0.10
Putamen	76.71 ± 33.54	73.62 ± 30.35	83.75 ± 32.05^†^	85.90 ± 31.65^†^	0.02	0.91	0.53	0.11
Cerebellum	70.92 ± 37.02	63.24 ± 42.46	67.93 ± 34.16	66.74 ± 39.11	0.96	0.38	0.52	0.00

*Note*: Data are the mean ± SD. Data reflects *n* = 15. Condition (*C*) = exercise vs. rest. Time (*T*) = insulin vs. fasting. η^2^ is reflective of a main effect of condition.

Abbreviations: *C* × *T*, condition by rime; INI, intranasal insulin.

*Compared with 0 min rest, *P* = 0.046.

^†^Condition effect, *P* < 0.05.

**TABLE 4:** Insulin‐stimulated cerebral blood flow after rest and exercise.

Brain region	Rest	Exercise	*P*‐value	Cohen's *d*
Left region, mL/min				
Hippocampus	−8.24 ± 31.90	3.60 ± 12.60	0.18	0.46
Caudate	0.38 ± 21.77	2.70 ± 13.28	0.67	0.11
Pallidum	−13.76 ± 26.91	1.43 ± 17.42	0.057	0.59
Putamen	−9.74 ± 33.59	2.53 ± 13.63	0.16	0.45
Cerebellum	−12.18 ± 25.33	−1.85 ± 14.49	0.19	0.44
Right region, mL/min				
Hippocampus	−3.74 ± 30.74	−0.91 ± 9.44	0.73	0.38
Caudate	−1.07 ± 18.22	−1.28 ± 9.74	0.96	0.01
Pallidum	−8.67 ± 22.12	4.18 ± 14.91	0.054	0.59
Putamen	−7.38 ± 31.15	2.15 ± 11.46	0.22	0.38
Cerebellum	−9.41 ± 26.45	−1.19 ± 10.86	0.29	0.38

*Note*: Data are the mean ± SD. Data reflect *n* = 15. Insulin‐stimulated was defined as post‐intranasal insulin minus fasting cerebral blood flow. *P*‐values were co‐varied to fasting CBF.

### 3.4 Correlations

**FIGURE 1:**
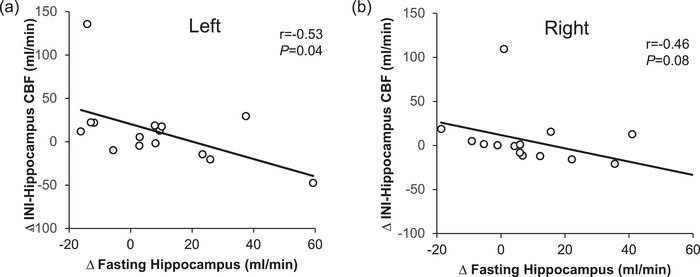
Fasting hippocampus CBF in relationship to INI‐stimulated CBF after exercise. Note that Pearson correlations were used to assess the change (Δ) in both fasting and INI‐stimulated CBF (i.e., exercise minus rest conditions). Abbreviations: CBF, cerebral blood flow; INI, intranasal insulin.

**FIGURE 2:**
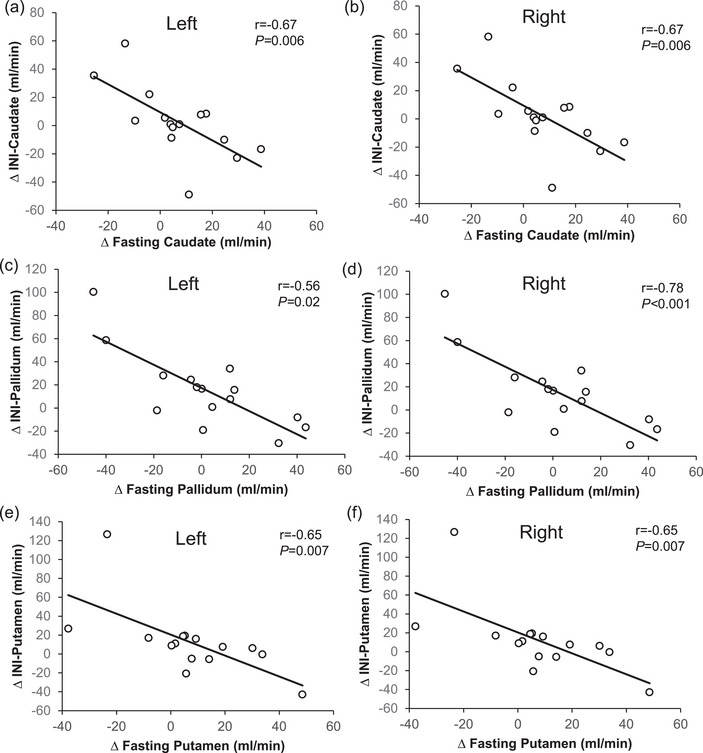
Fasting caudate nucleus (a, b), pallidum (c, d) and putamen (e, f) CBF in relationship to intranasal insulin‐stimulated CBF after exercise. Note that Pearson correlations were used to assess the change (Δ) in both fasting and INI‐stimulated CBF (i.e., exercise minus rest conditions). Abbreviations: CBF, cerebral blood flow; INI, intranasal insulin.

**FIGURE 3:**
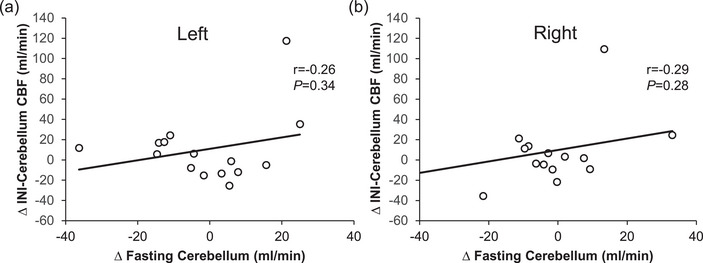
Fasting cerebellum CBF in relationship to intranasal insulin‐stimulated CBF after exercise. Note that Pearson correlations were used to assess the change (Δ) in both fasting and INI‐stimulated CBF (i.e., exercise minus rest conditions). Abbreviations: CBF, cerebral blood flow; INI, intranasal insulin.

The exercise‐induced increase in fasting CBF of the hippocampus (left, *r* = −0.53, *P* = 0.04; right, *r* = −0.46, *P* = 0.08; Figure 1), caudate (left, *r* = −0.67, *P* = 0.006; right, *r* = −0.81, *P* < 0.001), pallidum (left, *r* = −0.78, *P* < 0.001; right, *r* = −0.56, *P* = 0.02) and putamen (left, *r* = −0.65, *P* = 0.007; right, *r* = −0.73, *P* = 0.001; Figure 2), but not cerebellum (left, *r* = 0.26, *P* = 0.34; right, *r* = 0.29, *P* = 0.28, Figure 3), related to decreased CBF in response to INI. Age tended to be correlated with increased fasting CBF in the left pallidum (*r* = 0.58, *P* = 0.02) but not the right pallidum (*r* = 0.35, *P* = 0.19) after exercise. Age was also related to changes in INI‐stimulated putamen CBF of left (*r* = −0.43, *P* = 0.10), but not right (*r* = −0.35, *P* = 0.19) hemispheres, in addition to cerebellum (*r* = −0.45, *P* = 0.09) CBF of the left, but not right (*r* = −0.33, *P* = 0.22), hemispheres after exercise. Triglyceride levels were associated with age (*r* = −0.69, *P* = 0.004) and with exercise‐mediated changes in fasting hippocampus (left, *r* = −0.55, *P* = 0.03; right, *r* = −0.51, *P* = 0.04) CBF only. Elevated haemoglobin A1c tended to be correlated with fasting putamen CBF after exercise (left, *r* = 0.45, *P* = 0.09; right, *r* = 0.50, *P* = 0.056) only.

## 4 DISCUSSION

The primary finding of this pilot study was that a single bout of exercise performed the night before neuroimaging increased CBF in the hippocampus, putamen and pallidum. Although prior work has documented effects of acute exercise on CBF immediately following exercise (Palmer et al., 2023; Steventon et al., 2020), the present results suggest that some increases persist ∼14 h later. This effect appears to be attributable to increased fasting CBF, because only a trend emerged for exercise to increase pallidum CBF in response to INI after co‐varying for fasting CBF with medium effect sizes. Although we did not observe significant group effects of INI on CBF, there might be effects at the individual level. The increase in fasting pallidum CBF, among other regions, was correlated with lower INI‐stimulated CBF. These findings highlight that a moderate‐ to high‐intensity exercise bout might not only increase fasting CBF the next day but also alter brain insulin responsiveness in some adults with excess body weight. Such decreases, when they occur, can be viewed in light of findings reporting that ageing adults with excess weight, but without type 2 diabetes, had declines in INI‐stimulated accumbens CBF following diet‐induced weight loss (Tiedemann et al., 2022). Likewise, mixed nut consumption for 16 weeks was shown, in older adults who were overweight, to lower CBF insulin responses to INI in various right or left hemisphere regions independent of peripheral insulin sensitivity (Nijssen, Mensink, Plat et al., 2023). Given that ageing adults have elevated INI‐stimulated CBF relative to younger adults (Akintola et al., 2017), the present work suggests that lowering INI‐stimulated CBF might be a measure of improved brain insulin responsiveness. However, the only other study to date examining exercise‐mediated changes in CBF following INI (at 160 IU) reported increased INI‐stimulated CBF in the putamen among sedentary, young adults with obesity (Kullmann et al., 2022). Although this latter effect was probably mediated by peripheral fat mass loss to some extent, particularly visceral adipose tissue (Kullmann et al., 2022, 2020), it is worth noting that the putamen and pallidum are part of the basal ganglia and collectively play roles in sensorimotor control and in motivation/reward learning. The exact meaning behind these prior changes in CBF after exercise compared with other studies with diet is difficult to reconcile, but the study population (young vs. old) is one possibility. Regardless, the collective findings warrant additional attention, because these brain regions are implicated in memory, motor control, executive function and learning/motivation. Furthermore, these results are consistent with our work here, in that a single bout of exercise performed the night before might increase blood flow to these areas (i.e., hippocampus, putamen and pallidum) to support function prior to weight/fat loss. Moreover, our findings are similar to others who reported that a single, 20 min bout of exercise at 50%–70% heart rate reserve on an upright cycle ergometer increased fasting CBF in healthy men by ∼10%–12% in the hippocampus up to 60 min following exercise (Steventon et al., 2020). Furthermore, 15 min of moderate‐intensity aerobic exercise at 45%–55% heart rate reserve was found to increase global and region‐specific CBF in the 24 min after exercise among older adults, and this change was most profound in the hippocampus (Palmer et al., 2023). Thus, these data add to the literature by showing that an evening bout of exercise increases CBF the next morning, before weight loss or fitness gains.

Ageing adults and/or people with prediabetes are often reported to have lower fasting CBF and glucose metabolism compared with control subjects (Heni et al., 2014; Hirvonen et al., 2011; Honkala et al., 2018). This also has been shown to coincide with reduced insulin signalling, as evident from the brain of patients with Alzheimer's disease (Arnold et al., 2018). However, people at risk for Alzheimer's disease have elevated brain insulin‐stimulated CBF and/or glucose metabolism compared with control subjects (Heni et al., 2014; Hirvonen et al., 2011; Honkala et al., 2018). This insulin‐stimulated response in the brain might seem paradoxical at first, given that insulin‐stimulated skeletal muscle blood flow would be favourable. However, this elevated CBF response to insulin in at‐risk populations might be a compensatory mechanism to provide glucose as a fuel to neurons relative to lean control subjects, who respond near maximally to insulin in fasting states (Hirvonen et al., 2011). This aligns with views that the hippocampus, cerebral cortex and cerebellum, among other regions, have Glucose Transporter Protein Type‐4 (GLUT‐4 transporters) that are acted upon by insulin via the canonical signalling protein, Akt (Protein Kinase B) (Arnold et al., 2018). At the same time, the BBB becomes, in part, a limiting factor in allowing adequate peripheral insulin into the brain to carry out appropriate actions, because people with obesity often develop hyperinsulinaemia, and this hyperinsulinaemia saturates the BBB such that less insulin is found in the CSF (Rhea & Banks, 2019; Watson et al., 2022). In fact, ageing is related to lower circulating cortical insulin concentrations prior to dementia (Frölich et al., 1998). To bypass this potential variation in BBB integrity versus peripheral approaches to raise insulin availability (e.g., glucose ingestion or intravenous insulin infusion), we tested directly how INI acts on brain vasculature in ageing adults. Interestingly, we observed modest correlations, albeit not statistically significant, between age and the response to exercise on CBF measures, particularly with left hemisphere outcomes. These findings suggest that older adults might have increased fasting CBF in addition to brain insulin responsiveness following exercise compared with middle‐aged adults. Given the sample size, however, these present data ought to be interpreted with caution. In fact, no condition effect was noted for insulin‐stimulated CBF, and only the pallidum tended to change with INI stimulation after exercise. Nonetheless, these findings are consistent with prior exercise work (Honkala et al., 2018) and potential autoregulation. Indeed, the literature shows that older adults have differential CBF responses to INI compared with younger adults (Akintola et al., 2017). Here, we extend this concept that exercise might restore ‘CBF flexibility’ in middle‐aged to older adults, as observed in lean individuals (i.e., high fasting CBF and low insulin‐stimulated CBF). Larger exercise and mechanistic studies, however, await investigation to test this hypothesis in ageing adults.

Another consideration is that ageing adults commonly have elevated metabolic risk factors, such as hyperglycaemia and dyslipidaemia. We did not observe in this analysis a consistent relationship between haemoglobin A1c and the change in fasting CBF or INI‐stimulated CBF. However, age was inversely associated with lower triglyceride concentrations in the present study. This suggests that lipids could play some role in influencing the impact of exercise on CBF in our middle‐aged to older adults. This is of clinical interest, because triglycerides can promote atherosclerosis in conduit arteries leading to the brain, thereby reducing CBF (Meyer et al., 1987). In fact, triglycerides can impact the uptake of several hormones, including insulin, across the BBB (Urayama & Banks, 2008) and ultimately limit insulin availability and action. Moreover, circulating triglycerides are closely tied to both cognitive and executive function impairment (Farr et al., 2008; Frazier et al., 2015; Luchsinger & Mayeux, 2004). Clinically, it is important to recognize, however, that cognition was not affected by exercise in the present study. Although this is not necessarily surprising, given that our participants had normal cognitive function according to our inclusion criteria (i.e., Montreal Cognitive Assessment scores), our work suggests alterations in CBF regions tied to memory (e.g., hippocampus), and further work is needed to understand how exercise might contribute to preservation of cognition over time.

From a public health perspective, it is important to recognize that most studies testing the effect of INI on brain insulin action use 160 IU of insulin versus the clinical dose of 40 IU. Using such doses provides a supraphysiological insulin exposure to the brain and can promote spillover effects of insulin from the brain into the periphery and influence systemic blood pressure (Nijssen et al., 2023), in addition to CBF and the BBB (Gray et al., 2014; Kern et al., 2006). Importantly, we observed that administering INI had no effects on glucose or insulin, nor did it promote hypoglycaemia. This is significant, because prior work suggests that higher doses of INI prior to exercise raise the risk for hypoglycaemia (Gwizdala et al., 2021). Nevertheless, additional work is needed to assess how exercise might interact with INI to influence glucose and vascular regulation to ensure safety when combining INI with exercise.

This study has limitations. We used a relatively small sample size composed mostly of women who were White, educated, cognitively normal and right‐hand dominant individuals for this pilot study. As a result, generalizing these findings to others might be difficult. Our population might help to explain the strength of relationships in some outcomes, owing to right‐handed individuals often being left hemisphere dominant, in addition to the lack of change in cognitive outcomes. Furthermore, we are potentially underpowered owing to the nature of this pilot work, and we did not correct for multiple comparisons. We accept an α‐level of 0.05 as being statistically significant. Although effect size results support overall medium to large effects of exercise on CBF in the hippocampus, pallidum and putamen, caution should be used when interpreting our overall findings. By design, this study tested whether a single bout of aerobic exercise impacts CBF before and after INI, and we did not use a time‐matched control or an intranasal placebo control to discern independent effects of insulin. Furthermore, we did not measure arterial CO_2_ during the protocol, which might have influenced CBF measures. We elected not to assess CO_2_ because prior work using the same exercise model in a similar cohort of individuals indicated that there was no effect on respiratory gas (i.e., O_2_ uptake and CO_2_ output) derived fuel use (Heiston et al., 2021). However, this should be considered in future work to assess such potential mechanistic influences of insulin and CO_2_ vascular reactivity. Furthermore, CBF measures herein were not adjusted to beat‐to‐beat blood pressure throughout imaging. We previously reported no effect on fasting blood pressures using the same single‐bout exercise protocol performed the night before (Heiston et al., 2022). Although it is possible that blood pressure changes occurred post‐INI, the relevance of such an effect on CBF is unclear, given that recent cerebral autoregulation work suggests that a nearly 10–15 mmHg shift in mean arterial pressure is needed to alter CBF (Brassard et al., 2021). Regardless, additional work assessing such mechanisms is needed. Whether different intensities and/or modes (e.g., weightlifting) impact INI‐mediated CBF is also unknown. To this end, we elected to study CBF responses to insulin, although there is a lack of clear consensus on defining brain insulin sensitivity (e.g., PET vs. CBF). Recently, neuronal extracellular vesicles (nEVs) have garnered attention because they contain signalling proteins for neuronal cellular metabolism and insulin signalling, and they are upheld as a ‘liquid biopsy’ of the brain (Cleary et al., 2025; Delgado‐Peraza et al., 2023; Kapogiannis et al., 2019; Mustapic et al., 2019). Notably, 2 weeks of exercise at ∼70% of peak HR in older adults with prediabetes had no effect on fasting nEV insulin signalling proteins, although it increased glucose‐stimulated nEV‐derived total Akt after 60 min of a 75 g oral glucose tolerance test (Malin et al., 2025). Whether an acute bout of exercise modifies nEV insulin signalling remains to be seen. Lastly, food was not refed after exercise. Whether this energy deficit induced by exercise contributes to CBF changes is an area of work that awaits investigation.

## 5 CONCLUSION

In conclusion, we used INI to test brain insulin sensitivity via CBF with rigorous MRI protocols in a clinically relevant population. In this pilot study, we report that exercise increased CBF to the hippocampus, putamen and pallidum in particular. Although there was not a robust increase in CBF across regions studied herein after INI stimulation, it is noteworthy that the increase in fasting CBF was inversely associated with INI‐stimulated CBF. This suggests that exercise might promote coordinated changes in CBF between fasting and insulin‐stimulated states. Additional work is needed to examine how exercise might enhance brain insulin responsiveness to prevent, treat and/or manage the risk of cognitive decline in at‐risk populations.

## AUTHOR CONTRIBUTIONS

Steven K. Malin conceptualized the study design and hypotheses. Steven K. Malin, Ankit M. Shah, Michal S. Berri, David H. Zald and Daniel J. Battillo contributed to recruitment, data collection, data analysis and/or interpretation. Daniel J. Battillo and Steven K. Malin were responsible for statistical analysis. Steven K. Malin wrote the manuscript. All authors provided editorial comments, approved the final manuscript and agree to be accountable for all aspects of the work in ensuring that questions related to the accuracy or integrity of any part of the work are appropriately investigated and resolved. All persons designated as authors qualify for authorship, and all those who qualify for authorship are listed.

## CONFLICT OF INTEREST

None declared.

## Data Availability

These data have not been made publicly available. However, the corresponding author can provide further information on the data upon reasonable request.

## References

[eph70173-bib-0001] Adebimpe, A. , Bertolero, M. , Dolui, S. , Cieslak, M. , Murtha, K. , Baller, E. B. , Boeve, B. , Boxer, A. , Butler, E. R. , Cook, P. , Colcombe, S. , Covitz, S. , Davatzikos, C. , Davila, D. G. , Elliott, M. A. , Flounders, M. W. , Franco, A. R. , Gur, R. E. , Gur, R. C. , … Satterthwaite, T. D. (2022). ASLPrep: A platform for processing of arterial spin labeled MRI and quantification of regional brain perfusion. Nature Methods, 19(6), 683–686. 35689029 10.1038/s41592-022-01458-7 PMC10548890

[eph70173-bib-0002] Akintola, A. A. , van Opstal, A. M. , Westendorp, R. G. , Postmus, I. , van der Grond, J. , & van Heemst, D. (2017). Effect of intranasally administered insulin on cerebral blood flow and perfusion; a randomized experiment in young and older adults. Aging (Albany NY), 9(3), 790–802. 28291957 10.18632/aging.101192 PMC5391232

[eph70173-bib-0003] Arnold, S. E. , Arvanitakis, Z. , Macauley‐Rambach, S. L. , Koenig, A. M. , Wang, H. Y. , Ahima, R. S. , Craft, S. , Gandy, S. , Buettner, C. , Stoeckel, L. E. , Holtzman, D. M. , & Nathan, D. M. (2018). Brain insulin resistance in type 2 diabetes and Alzheimer disease: Concepts and conundrums. Nature Reviews Neurology, 14(3), 168–181. 29377010 10.1038/nrneurol.2017.185 PMC6098968

[eph70173-bib-0004] Bendlin, B. B. , Carlsson, C. M. , Gleason, C. E. , Johnson, S. C. , Sodhi, A. , Gallagher, C. L. , Puglielli, L. , Engelman, C. D. , Ries, M. L. , Xu, G. , Wharton, W. , & Asthana, S. (2010). Midlife predictors of Alzheimer's disease. Maturitas, 65(2), 131–137. 20044221 10.1016/j.maturitas.2009.12.014 PMC2895971

[eph70173-bib-0005] Brassard, P. , Labrecque, L. , Smirl, J. D. , Tymko, M. M. , Caldwell, H. G. , Hoiland, R. L. , Lucas, S. J. E. , Denault, A. Y. , Couture, E. J. , & Ainslie, P. N. (2021). Losing the dogmatic view of cerebral autoregulation. Physiological Reports, 9(15), e14982. 34323023 10.14814/phy2.14982 PMC8319534

[eph70173-bib-0006] Brown, C. , Pemberton, S. , Babin, A. , Abdulhameed, N. , Noonan, C. , Brown, M. B. , Banks, W. A. , & Rhea, E. M. (2022). Insulin blood‐brain barrier transport and interactions are greater following exercise in mice. Journal of Applied Physiology (1985), 132(3), 824–834. 10.1152/japplphysiol.00866.2021 PMC8917914 35175106

[eph70173-bib-0007] Cleary, J. A. , Kumar, A. , Craft, S. , & Deep, G. (2025). Neuron‐derived extracellular vesicles as a liquid biopsy for brain insulin dysregulation in Alzheimer's disease and related disorders. Alzheimer's & Dementia, 21(2), e14497. 10.1002/alz.14497 PMC11848159 39822132

[eph70173-bib-0008] Cooper, C. , Sommerlad, A. , Lyketsos, C. G. , & Livingston, G. (2015). Modifiable predictors of dementia in mild cognitive impairment: A systematic review and meta‐analysis. American Journal of Psychiatry, 172(4), 323–334. 25698435 10.1176/appi.ajp.2014.14070878

[eph70173-bib-0009] Delgado‐Peraza, F. , Nogueras‐Ortiz, C. , Simonsen, A. H. , Knight, D. D. , Yao, P. J. , Goetzl, E. J. , Jensen, C. S. , Høgh, P. , Gottrup, H. , Vestergaard, K. , Hasselbalch, S. G. , & Kapogiannis, D. (2023). Neuron‐derived extracellular vesicles in blood reveal effects of exercise in Alzheimer's disease. Alzheimer's Research & Therapy, 15(1), 156–159. 10.1186/s13195-023-01303-9 PMC10510190 37730689

[eph70173-bib-0010] Eisenmenger, L. B. , Peret, A. , Famakin, B. M. , Spahic, A. , Roberts, G. S. , Bockholt, J. H. , Johnson, K. M. , & Paulsen, J. S. (2023). Vascular contributions to Alzheimer's disease. Translational Research, 254, 41–53. 36529160 10.1016/j.trsl.2022.12.003 PMC10481451

[eph70173-bib-0011] Farr, S. A. , Yamada, K. A. , Butterfield, D. A. , Abdul, H. M. , Xu, L. , Miller, N. E. , Banks, W. A. , & Morley, J. E. (2008). Obesity and hypertriglyceridemia produce cognitive impairment. Endocrinology, 149(5), 2628–2636. 18276751 10.1210/en.2007-1722 PMC2329289

[eph70173-bib-0012] Frazier, D. T. , Bettcher, B. M. , Dutt, S. , Patel, N. , Mungas, D. , Miller, J. , Green, R. , & Kramer, J. H. (2015). Relationship between insulin‐resistance processing speed and specific executive function profiles in neurologically intact older adults. Journal of the International Neuropsychological Society, 21(8), 622–628. 26272269 10.1017/S1355617715000624 PMC4764989

[eph70173-bib-0013] Frölich, L. , Blum‐Degen, D. , Bernstein, H. G. , Engelsberger, S. , Humrich, J. , Laufer, S. , Muschner, D. , Thalheimer, A. , Türk, A. , Hoyer, S. , Zöchling, R. , Boissl, K. W. , Jellinger, K. , & Riederer, P. (1998). Brain insulin and insulin receptors in aging and sporadic Alzheimer's disease. Journal of Neural Transmission (Vienna), 105, 423–438. 10.1007/s007020050068 9720972

[eph70173-bib-0014] Gottesman, R. F. , Albert, M. S. , Alonso, A. , Coker, L. H. , Coresh, J. , Davis, S. M. , Deal, J. A. , McKhann, G. M. , Mosley, T. H. , Sharrett, A. R. , Schneider, A. L. C. , Windham, B. G. , Wruck, L. M. , & Knopman, D. S. (2017). Associations between midlife vascular risk factors and 25‐year incident dementia in the atherosclerosis risk in communities (ARIC) Cohort. Journal of the American Medical Association Neurology, 74(10), 1246. 28783817 10.1001/jamaneurol.2017.1658 PMC5710244

[eph70173-bib-0015] Gray, S. M. , Meijer, R. I. , & Barrett, E. J. (2014). Insulin regulates brain function, but how does it get there? Diabetes, 63(12), 3992–3997. 25414013 10.2337/db14-0340 PMC4237995

[eph70173-bib-0016] Gwizdala, K. L. , Ferguson, D. P. , Kovan, J. , Novak, V. , & Pontifex, M. B. (2021). Placebo controlled phase II clinical trial: Safety and efficacy of combining intranasal insulin & acute exercise. Metabolic Brain Disease, 36(6), 1289–1303. 33856613 10.1007/s11011-021-00727-2

[eph70173-bib-0017] Heiston, E. M. , Ballantyne, A. , La Salvia, S. , Musante, L. , Erdbrügger, U. , & Malin, S. K. (2022). Acute exercise decreases insulin‐stimulated extracellular vesicles in conjunction with augmentation index in adults with obesity. The Journal of Physiology, 601(22), 5033–5050. 35081660 10.1113/JP282274 PMC9314457

[eph70173-bib-0018] Heiston, E. M. , Gilbertson, N. M. , Eichner, N. Z. M. , & Malin, S. K. (2021). A low‐calorie diet with or without exercise reduces postprandial aortic waveform in females with obesity. Medicine and Science in Sports and Exercise, 53(4), 796–803. 32925495 10.1249/MSS.0000000000002515

[eph70173-bib-0019] Heiston, E. M. , Liu, Z. , Ballantyne, A. , Kranz, S. , & Malin, S. K. (2021). A single bout of exercise improves vascular insulin sensitivity in adults with obesity. Obesity (Silver Spring), 29(9), 1487–1496. 34339111 10.1002/oby.23229 PMC8387339

[eph70173-bib-0020] Heiston, E. M. , & Malin, S. K. (2019). Impact of exercise on inflammatory mediators of metabolic and vascular insulin resistance in type 2 diabetes. Advances in Experimental Medicine and Biology, 1134, 271–294. 30919343 10.1007/978-3-030-12668-1_15

[eph70173-bib-0021] Heni, M. , Wagner, R. , Kullmann, S. , Veit, R. , Mat Husin, H. , Linder, K. , Benkendorff, C. , Peter, A. , Stefan, N. , Häring, H. U. , Preissl, H. , & Fritsche, A. (2014). Central insulin administration improves whole‐body insulin sensitivity via hypothalamus and parasympathetic outputs in men. Diabetes, 63(12), 4083–4088. 25028522 10.2337/db14-0477

[eph70173-bib-0022] Hirvonen, J. , Virtanen, K. A. , Nummenmaa, L. , Hannukainen, J. C. , Honka, M. , Bucci, M. , Nesterov, S. V. , Parkkola, R. , Rinne, J. , Iozzo, P. , & Nuutila, P. (2011). Effects of insulin on brain glucose metabolism in impaired glucose tolerance. Diabetes, 60(2), 443–447. 21270256 10.2337/db10-0940 PMC3028343

[eph70173-bib-0023] Honkala, S. M. , Johansson, J. , Motiani, K. K. , Eskelinen, J. J. , Virtanen, K. A. , Löyttyniemi, E. , Knuuti, J. , Nuutila, P. , Kalliokoski, K. K. , & Hannukainen, J. C. (2018). Short‐term interval training alters brain glucose metabolism in subjects with insulin resistance. Journal of Cerebral Blood Flow and Metabolism, 38(10), 1828–1838. 28959911 10.1177/0271678X17734998 PMC6168908

[eph70173-bib-0024] Huang, C. C. , Rolls, E. T. , Feng, J. , & Lin, C. P. (2022). An extended Human Connectome Project multimodal parcellation atlas of the human cortex and subcortical areas. Brain Structure and Function, 227(3), 763–778. 34791508 10.1007/s00429-021-02421-6

[eph70173-bib-0025] Kapogiannis, D. , Mustapic, M. , Shardell, M. D. , Berkowitz, S. T. , Diehl, T. C. , Spangler, R. D. , Tran, J. , Lazaropoulos, M. P. , Chawla, S. , Gulyani, S. , Eitan, E. , An, Y. , Huang, C. , Oh, E. S. , Lyketsos, C. G. , Resnick, S. M. , Goetzl, E. J. , & Ferrucci, L. (2019). Association of extracellular vesicle biomarkers with Alzheimer disease in the Baltimore longitudinal study of aging. Journal of the American Medical Association Neurology, 76(11), 1340. 31305918 10.1001/jamaneurol.2019.2462 PMC6632160

[eph70173-bib-0026] Katsel, P. , Roussos, P. , Beeri, M. S. , Gama‐Sosa, M. A. , Gandy, S. , Khan, S. , & Haroutunian, V. (2018). Parahippocampal gyrus expression of endothelial and insulin receptor signaling pathway genes is modulated by Alzheimer's disease and normalized by treatment with anti‐diabetic agents. PLoS ONE, 13(11), e0206547. 30383799 10.1371/journal.pone.0206547 PMC6211704

[eph70173-bib-0027] Kern, W. , Benedict, C. , Schultes, B. , Plohr, F. , Moser, A. , Born, J. , Fehm, H. L. , & Hallschmid, M. (2006). Low cerebrospinal fluid insulin levels in obese humans. Diabetologia, 49(11), 2790–2792. 16951936 10.1007/s00125-006-0409-y

[eph70173-bib-0028] Kullmann, S. , Goj, T. , Veit, R. , Fritsche, L. , Wagner, L. , Schneeweiss, P. , Hoene, M. , Hoffmann, C. , Machann, J. , Niess, A. , Preissl, H. , Birkenfeld, A. L. , Peter, A. , Häring, H. U. , Fritsche, A. , Moller, A. , Weigert, C. , & Heni, M. (2022). Exercise restores brain insulin sensitivity in sedentary adults who are overweight and obese. Journal of Clinical Investigation Insight, 7(18), e161498. 36134657 10.1172/jci.insight.161498 PMC9675563

[eph70173-bib-0029] Kullmann, S. , Valenta, V. , Wagner, R. , Tschritter, O. , Machann, J. , Häring, H. U. , Preissl, H. , Fritsche, A. , & Heni, M. (2020). Brain insulin sensitivity is linked to adiposity and body fat distribution. Nature Communications, 11(1), 1841–y. 10.1038/s41467-020-15686-y PMC7160151 32296068

[eph70173-bib-0030] Kullmann, S. , Veit, R. , Peter, A. , Pohmann, R. , Scheffler, K. , Häring, H. , Fritsche, A. , Preissl, H. , & Heni, M. (2018). Dose‐dependent effects of intranasal insulin on resting‐state brain activity. Journal of Clinical Endocrinology and Metabolism, 103(1), 253–262. 29095982 10.1210/jc.2017-01976

[eph70173-bib-0031] Lake, S. L. , Guadagni, V. , Kendall, K. D. , Chadder, M. , Anderson, T. J. , Leigh, R. , Rawling, J. M. , Hogan, D. B. , Hill, M. D. , & Poulin, M. J. (2022). Aerobic exercise training in older men and women‐cerebrovascular responses to submaximal exercise: Results from the brain in motion study. Physiological Reports, 10(4), e15158. 35212167 10.14814/phy2.15158 PMC8874289

[eph70173-bib-0032] Leclerc, M. , Bourassa, P. , Tremblay, C. , Caron, V. , Sugère, C. , Emond, V. , Bennett, D. A. , & Calon, F. (2022). Cerebrovascular insulin receptors are defective in Alzheimer's disease. Brain, 146(1), 75–90. 10.1093/brain/awac309 PMC9897197 36280236

[eph70173-bib-0033] Luchsinger, J. A. , & Mayeux, R. (2004). Cardiovascular risk factors and Alzheimer's disease. Current Atherosclerosis Reports, 6(4), 261–266. 15191699 10.1007/s11883-004-0056-z

[eph70173-bib-0034] Makino, K. , Lee, S. , Bae, S. , Chiba, I. , Harada, K. , Katayama, O. , Shinkai, Y. , Makizako, H. , & Shimada, H. (2021). Diabetes and prediabetes inhibit reversion from mild cognitive impairment to normal cognition. Journal of the American Medical Directors Association, 22(9), 1912–1918.e2. 33798483 10.1016/j.jamda.2021.02.033

[eph70173-bib-0035] Malin, S. K. , Battillo, D. J. , Beeri, M. S. , Mustapic, M. , Delgado‐Peraza, F. , & Kapogiannis, D. (2025). Two weeks of exercise alters neuronal extracellular vesicle insulin signaling proteins and pro‐BDNF in older adults with prediabetes. Aging Cell, 24(1), e14369. 39421964 10.1111/acel.14369 PMC11709104

[eph70173-bib-0036] Malin, S. K. , Rynders, C. A. , Weltman, J. Y. , Barrett, E. J. , & Weltman, A. (2016). Exercise intensity modulates glucose‐stimulated insulin secretion when adjusted for adipose, liver and skeletal muscle insulin resistance. PLoS ONE, 11(4), e0154063. 27111219 10.1371/journal.pone.0154063 PMC4844153

[eph70173-bib-0037] Malin, S. K. , Stewart, N. R. , Ude, A. A. , & Alderman, B. L. (2022). Brain insulin resistance and cognitive function: Influence of exercise. Journal of Applied Physiology (1985), 133(6), 1368–1380. 10.1152/japplphysiol.00375.2022 PMC9744647 36269295

[eph70173-bib-0038] Marosi, K. , Bori, Z. , Hart, N. , Sárga, L. , Koltai, E. , Radák, Z. , & Nyakas, C. (2012). Long‐term exercise treatment reduces oxidative stress in the hippocampus of aging rats. Neuroscience, 226, 21–28. 22982624 10.1016/j.neuroscience.2012.09.001

[eph70173-bib-0039] Meyer, J. S. , Rogers, R. L. , Mortel, K. F. , & Judd, B. W. (1987). Hyperlipidemia is a risk factor for decreased cerebral perfusion and stroke. Archives of Neurology, 44(4), 418–422. 3827697 10.1001/archneur.1987.00520160052014

[eph70173-bib-0040] Mustapic, M. , Tran, J. , Craft, S. , & Kapogiannis, D. (2019). Extracellular vesicle biomarkers track cognitive changes following intranasal insulin in Alzheimer's disease. Journal of Alzheimer's Disease, 69(2), 489–498. 10.3233/JAD-180578 PMC6668911 30958348

[eph70173-bib-0041] Nijssen, K. M. R. , Mensink, R. P. , & Joris, P. J. (2023). Effects of intranasal insulin administration on cerebral blood flow and cognitive performance in adults: A systematic review of randomized, placebo‐controlled intervention studies. Neuroendocrinology, 113(1), 1–13. 36219990 10.1159/000526717

[eph70173-bib-0041a] Nijssen, K. M. R. , Mensink, R. P. , Plat, J. , & Joris, P. J. (2023). Longer‐term mixed nut consumption improves brain vascular function and memory: A randomized, controlled crossover trial in older adults. Clinical Nutrition, 42(7), 1067–1075. 37296019 10.1016/j.clnu.2023.05.025

[eph70173-bib-0042] Novak, V. , Mantzoros, C. S. , Novak, P. , McGlinchey, R. , Dai, W. , Lioutas, V. , Buss, S. , Fortier, C. B. , Khan, F. , Aponte Becerra, L. , & Ngo, L. H. (2022). MemAID: Memory advancement with intranasal insulin vs. placebo in type 2 diabetes and control participants: A randomized clinical trial. Journal of Neurology, 269(9), 4817–4835. 35482079 10.1007/s00415-022-11119-6 PMC9046533

[eph70173-bib-0044] Ogino, E. , Manly, J. J. , Schupf, N. , Mayeux, R. , & Gu, Y. (2019). Current and past leisure time physical activity in relation to risk of Alzheimer's disease in older adults. Alzheimer's & Dementia, 15(12), 1603–1611. 10.1016/j.jalz.2019.07.013 PMC6948182 31587996

[eph70173-bib-0045] Ogoh, S. , Fadel, P. J. , Zhang, R. , Selmer, C. , Jans, Ø. , Secher, N. H. , & Raven, P. B. (2005). Middle cerebral artery flow velocity and pulse pressure during dynamic exercise in humans. American Journal of Physiology‐Heart and Circulatory Physiology, 288(4), H1526–H1531. 15591094 10.1152/ajpheart.00979.2004

[eph70173-bib-0046] Pal, K. , Mukadam, N. , Petersen, I. , & Cooper, C. (2018). Mild cognitive impairment and progression to dementia in people with diabetes, prediabetes and metabolic syndrome: A systematic review and meta‐analysis. Social Psychiatry and Psychiatric Epidemiology, 53(11), 1149–1160. 30182156 10.1007/s00127-018-1581-3 PMC6208946

[eph70173-bib-0047] Palmer, J. A. , Morris, J. K. , Billinger, S. A. , Lepping, R. J. , Martin, L. , Green, Z. , & Vidoni, E. D. (2023). Hippocampal blood flow rapidly and preferentially increases after a bout of moderate‐intensity exercise in older adults with poor cerebrovascular health. Cerebral Cortex, 33(9), 5297–5306. 36255379 10.1093/cercor/bhac418 PMC10152056

[eph70173-bib-0048] Park, H. , Park, S. , Kim, C. , Shin, M. , & Kim, T. (2019). Exercise alleviates cognitive functions by enhancing hippocampal insulin signaling and neuroplasticity in high‐fat diet‐induced obesity. Nutrients, 11(7), 1603. 31311133 10.3390/nu11071603 PMC6683269

[eph70173-bib-0050] Rhea, E. M. , & Banks, W. A. (2019). Role of the blood‐brain barrier in central nervous system insulin resistance. Frontiers in Neuroscience, 13, 521. 31213970 10.3389/fnins.2019.00521 PMC6558081

[eph70173-bib-0051] Ruegsegger, G. N. , Vanderboom, P. M. , Dasari, S. , Klaus, K. A. , Kabiraj, P. , McCarthy, C. B. , Lucchinetti, C. F. , & Nair, K. S. (2019). Exercise and metformin counteract altered mitochondrial function in the insulin‐resistant brain. Journal of Clinical Investigation Insight, 4(18), e130681. 31534057 10.1172/jci.insight.130681 PMC6795285

[eph70173-bib-0053] Sink, K. M. , Espeland, M. A. , Castro, C. M. , Church, T. , Cohen, R. , Dodson, J. A. , Guralnik, J. , Hendrie, H. C. , Jennings, J. , Katula, J. , Lopez, O. L. , McDermott, M. M. , Pahor, M. , Reid, K. F. , Rushing, J. , Verghese, J. , Rapp, S. , Williamson, J. D. , & LIFE Study Investigators . (2015). Effect of a 24‐month physical activity intervention vs health education on cognitive outcomes in sedentary older adults: The LIFE Randomized Trial. Journal of the American Medical Association, 314(8), 781. 26305648 10.1001/jama.2015.9617 PMC4698980

[eph70173-bib-0054] Steventon, J. J. , Foster, C. , Furby, H. , Helme, D. , Wise, R. G. , & Murphy, K. (2020). Hippocampal blood flow is increased after 20 min of moderate‐intensity exercise. Cerebral Cortex, 30(2), 525–533. 31216005 10.1093/cercor/bhz104 PMC7703728

[eph70173-bib-0055] Teresi, J. A. , Yu, X. , Stewart, A. L. , & Hays, R. D. (2022). Guidelines for designing and evaluating feasibility pilot studies. Medical Care, 60(1), 95–103. 34812790 10.1097/MLR.0000000000001664 PMC8849521

[eph70173-bib-0056] Tiedemann, L. J. , Meyhöfer, S. M. , Francke, P. , Beck, J. , Büchel, C. , & Brassen, S. (2022). Insulin sensitivity in mesolimbic pathways predicts and improves with weight loss in older dieters. eLife, 11, e76835. 36170006 10.7554/eLife.76835 PMC9519148

[eph70173-bib-0057] Tomoto, T. , Liu, J. , Tseng, B. Y. , Pasha, E. P. , Cardim, D. , Tarumi, T. , Hynan, L. S. , Munro Cullum, C. , & Zhang, R. (2021). One‐year aerobic exercise reduced carotid arterial stiffness and increased cerebral blood flow in amnestic mild cognitive impairment. Journal of Alzheimer's Disease, 80(2), 841–853. 10.3233/JAD-201456 33579857

[eph70173-bib-0058] Tomoto, T. , Verma, A. , Kostroske, K. , Tarumi, T. , Patel, N. R. , Pasha, E. P. , Riley, J. , Tinajero, C. D. , Hynan, L. S. , Rodrigue, K. M. , Kennedy, K. M. , Park, D. C. , & Zhang, R. (2023). One‐year aerobic exercise increases cerebral blood flow in cognitively normal older adults. Journal of Cerebral Blood Flow and Metabolism, 43(3), 404–418. 36250505 10.1177/0271678X221133861 PMC9941859

[eph70173-bib-0059] Trotier, A. J. , Dilharreguy, B. , Anandra, S. , Corbin, N. , Lefrançois, W. , Ozenne, V. , Miraux, S. , & Ribot, E. J. (2022). The compressed sensing MP2RAGE as a surrogate to the MPRAGE for neuroimaging at 3 T. Investigative Radiology, 57(6), 366–378. 35030106 10.1097/RLI.0000000000000849 PMC9390231

[eph70173-bib-0060] Urayama, A. , & Banks, W. A. (2008). Starvation and triglycerides reverse the obesity‐induced impairment of insulin transport at the blood‐brain barrier. Endocrinology, 149(7), 3592–3597. 18403490 10.1210/en.2008-0008 PMC2453080

[eph70173-bib-0061] Watson, L. S. , Wilken‐Resman, B. , Williams, A. , DiLucia, S. , Sanchez, G. , McLeod, T. L. , & Sims‐Robinson, C. (2022). Hyperinsulinemia alters insulin receptor presentation and internalization in brain microvascular endothelial cells. Diabetes and Vascular Disease Research, 19(4), 14791641221118626. 35975361 10.1177/14791641221118626 PMC9393688

[eph70173-bib-0062] Wingrove, J. , Swedrowska, M. , Scherließ, R. , Parry, M. , Ramjeeawon, M. , Taylor, D. , Gauthier, G. , Brown, L. , Amiel, S. , Zelaya, F. , & Forbes, B. (2019). Characterisation of nasal devices for delivery of insulin to the brain and evaluation in humans using functional magnetic resonance imaging. Journal of Controlled Release, 302, 140–147. 30953665 10.1016/j.jconrel.2019.03.032

[eph70173-bib-0063] Wingrove, J. O. , O'Daly, O. , Forbes, B. , Swedrowska, M. , Amiel, S. A. , & Zelaya, F. O. (2021). Intranasal insulin administration decreases cerebral blood flow in cortico‐limbic regions: A neuropharmacological imaging study in normal and overweight males. Diabetes, Obesity & Metabolism, 23(1), 175–185. 10.1111/dom.14213 33026175

